# Aging Research in Focus: Scholarly Output of the de Tornyay Center for Healthy Aging Network

**DOI:** 10.1093/geroni/igaf122.2388

**Published:** 2025-12-31

**Authors:** Wenting Peng, Paige Bartlett, Yanjing Liang, Priscilla Carmiol-Rodriguez, Basia Belza

**Affiliations:** University of Washington, Seattle, Washington, United States; University of Washington, Seattle, Washington, United States; University of Washington, Seattle, Washington, United States; University of Washington, Seattle, Washington, United States; University of Washington, Seattle, Washington, United States

## Abstract

Since 1998, the de Tornyay Center for Healthy Aging at the School of Nursing, University of Washington has served as a catalyst for promoting healthy aging research. The purpose of our project was to analyze publications authored by scholars with aging interests and affiliated or associated with the de Tornyay Center over the past five years. We searched Web of Science for publications authored by 41 faculty and scholars, published between January 1, 2020, and December 31, 2024. Conference abstracts were excluded. The R-package BibliometrixRuan was used to perform analyses. Among 480 identified publications, 394 were research articles, 48 were review articles, 25 were editorial material, 7 were letters to the editor, and 6 were corrections. The most frequently published journal was Biological Research for Nursing (N = 14). Overall, the publications had 2,221 co-authors and an average of 8.7 co-authors. International collaborations accounted for 28.0% of the publications. These works were cited in 4,849 articles, contributing to a total of 5,113 citations, with an average of 10.67 citations per publication. The H-index for these articles was 28, suggesting that 28 papers had each been cited at least 28 times. The most frequent keywords in the publications, with dementia being the most common, followed by HIV, irritable bowel syndrome, depression, and older adults. These findings highlight the de Tornyay Center’s impact in mentoring scholars and supporting innovative research addressing key aging challenges. The center’s interdisciplinary contributions reinforce its role in advancing knowledge and promoting healthy aging across the lifespan.

